# Personal Values, Health Beliefs, and the Use of Complementary, Alternative and Integrative Medicine in Finland

**DOI:** 10.1111/sjop.70104

**Published:** 2026-05-23

**Authors:** Salla Ahola, Maija Pyykkönen, Pauliina Aarva

**Affiliations:** ^1^ Faculty of Management and Business Tampere University Tampere Finland; ^2^ Socon—Social and Health Consultants Ltd Tampere Finland; ^3^ School of Humanities University of Eastern Finland Joensuu Finland

**Keywords:** complementary, alternative and integrative medicine (CAIM), Finland, health beliefs, personal values

## Abstract

This study examines the relationships of personal values and health beliefs with past and intended use of complementary, alternative and integrative medicine (CAIM) in Finland. It also investigates the extent to which CAIM users and non‐users endorse holistic versus reductionistic health beliefs and whether experiencing benefits or harms from CAIM use is related to users' intention to continue using CAIM. Data were drawn from the survey responses of 3244 Finns who were randomly selected from a commercial, probability‐based online panel. The results of logistic regression analyses indicated that scoring low on conservation (vs. openness to change) values, high on holistic health beliefs, and low on reductionistic health beliefs were associated with a higher likelihood of having used CAIM. High levels of holistic health beliefs and low levels of reductionistic health beliefs were associated with a higher likelihood of intending to use CAIM in the future or to continue CAIM use. Individuals who had used CAIM were more likely to intend to use it in the future than those who had not. CAIM users who had experienced benefits or had not experienced harms from CAIM use were more likely to intend to continue use. Both CAIM users and non‐users scored higher on holistic than on reductionistic health beliefs. The results highlight the role of health beliefs in past and intended future CAIM use and the relevance of experienced everyday effectiveness of CAIM for use intentions. This study demonstrates that a holistic conception of health and illness treatment is widely shared and not only a feature of CAIM users.

## Keypoints

- Conservation (vs. openness to change) values are negatively associated with past CAIM use.
- Holistic health beliefs are positively and reductionistic health beliefs are negatively associated with past and intended use of CAIM. However, both CAIM users and non‐users endorse holistic health beliefs more than reductionistic health beliefs, which indicates that a holistic conception of health and illness treatment is widely shared.
- Having experienced benefits from CAIM use is most strongly related to an intention to continue use, which points to the importance of the experienced everyday effectiveness of CAIM.

## 1 Introduction

Complementary, alternative and integrative medicine (CAIM) encompasses a wide range of products, therapies and practices used outside conventional medicine or together with it (Lee et al. 2022; Ng et al. 2022) to treat illness or to improve health and well‐being (Kristoffersen et al. 2021; Maghrabi et al. 2025; Tangkiatkumjai et al. 2020). The term *complementary* refers to “additional health care practices that are not part of a country's mainstream medicine” (World Health Organization [WHO] 2025, p. viii), and *alternative* refers to practices or approaches used in place of conventional medicine (National Center for Complementary and Integrative Health [NCCIH] 2021). *Integrative* medicine, in turn, combines “biomedical and traditional and/or complementary medical knowledge, skills and practices” (WHO 2025, p. vii). For clarity and consistency, we use the term *conventional medicine* throughout this article to refer to what is also described as *mainstream medicine*, *biomedicine*, or *Western medicine*, while acknowledging that these terms entail nuanced differences in meaning.

Although we were aware that the term complementary and alternative medicine (CAM; Wieland et al. 2011) is widely used in the scientific literature, we chose to use CAIM, which includes the term *integrative*, for three main reasons. First, CAIM was derived from a systematic review of terminology in research publications (Ng et al. 2022, 2023). Second, it reflects recent international developments, as the WHO explicitly included *integrative* medicine in its newly published *Global Traditional Medicine Strategy 2025–2034* (WHO 2025), thereby redefining traditional and complementary medicine (TCM; WHO 2013) as traditional, complementary and integrative medicine (TCIM; WHO 2025). Other related terms include traditional, complementary and alternative medicine (TCAM; Lee et al. 2022) and complementary and integrative health (CIH; Ho et al. 2021). Third, in our view, the term CAIM appropriately describes the situation in Finland, where this study was conducted: On the one hand, the terms *alternative treatments* and *complementary and alternative therapies* predominate in the official and health policy discourse in Finland (Ministry of Social Affairs and Health 2009; Publications of the Finnish Government 2023, 40), although in practice, CAIM modalities are more often used alongside conventional healthcare services than as alternatives (Kemppainen and Kemppainen 2025). On the other hand, several CAIM modalities have been integrated into the healthcare system. Licensed chiropractors, osteopaths and naprapaths are authorized healthcare professionals under Finnish law (Decree on Health Care Professionals 1994), practising predominantly as private practitioners. Moreover, acupuncture, hypnosis, relaxation therapies, meditation/mindfulness, manual therapies and yoga are referenced in physicians' evidence‐based *Current Care Guidelines* (kaypahoito.fi/en/) developed by the Finnish Medical Society Duodecim, which evaluates and discusses their potential as adjuncts within conventional clinical care.^1^

Although there is scientific evidence for the effectiveness of some CAIM practices, this evidence is generally limited (Brodin Danell et al. 2020; Ernst 2022; Hoenders et al. 2024; Hunter et al. 2026). Nevertheless, CAIM use is popular, with the estimated prevalence in general population samples ranging from 11.6% to 71.3% (Kemppainen and Kemppainen 2025; Lee et al. 2022) and reaching 51.1% in Finland (Pyykkönen et al. 2023) where the present study was conducted. However, there are differences across studies and countries regarding which therapies fall within the scope of CAIM (Ng et al. 2022). The main reasons for CAIM use include its perceived and expected benefits and safety as well as dissatisfaction with conventional medicine (Tangkiatkumjai et al. 2020). Beliefs, cognitions, and personality are associated with CAIM use (Abheiden et al. 2020; Bishop et al. 2007; Galbraith et al. 2018).

This study aims to gain further insight into what motivates people to use CAIM. We examine the relationships of personal values and health beliefs with CAIM use and future use intentions among Finnish people. We also seek to determine whether CAIM users' experienced benefits and harms from CAIM use play a role in their intention to continue use and whether personal values and health beliefs contribute to this intention. As these factors have not been analyzed simultaneously in previous studies, we aim to shed light on their relative importance in the context of CAIM use. Furthermore, incorporating participants' intention to use CAIM allows us to include a future perspective that, to our knowledge, has not been examined in relation to either personal values or health beliefs, except for single studies in specific contexts of hypothetical symptom scenarios and food supplement use (Ahola 2020; Aschemann‐Witzel and Grunert 2015). Finally, we consider not only holistic health beliefs, which have been linked with CAIM use (Bishop et al. 2007; Tangkiatkumjai et al. 2020), but also reductionistic health beliefs, which have been conceptually connected with conventional medicine (Graham et al. 2021) but have not been examined empirically. We operationalize and measure reductionistic health beliefs for the first time and compare the extent to which CAIM users and non‐users endorse holistic versus reductionistic health beliefs.

### 1.1 Personal Values

Human and personal values, typically considered “abstract ideals that guide human choices and behavior” (Pincus 2024, 1706) have only occasionally been considered in the context of CAIM use (Abheiden et al. 2020; Ahola 2020; Messerli‐Rohrbach 2000) and in health behaviors more generally (e.g., Aavik et al. 2014; Park et al. 2026). The concept of values in studies referring to patient values often employs this term without a clear conceptual definition or operationalisation, uses it interchangeably with other terms such as attitudes and beliefs (Karimi‐Dehkordi et al. 2019), or applies it in a sense that does not refer to values as abstract ideals but rather to practical or psychological value (i.e., benefits or utility) for individuals as consumers of a specific health care “brand,” such as CAIM. An example of the latter is Sirois et al.'s (2016) consumer commitment model (CCM), in which utilitarian values refer to various experienced physiological or psychological benefits of CAIM, whereas symbolic values refer to a perceived congruency or ‘fit’ between an individual's belief system and CAIM as a consumer brand. In this study, we conceptualize values using Schwartz's (1992) theory of basic human values, a leading personal values framework (Lechner et al. 2024) that covers both the content and structure of values. In addition, we use CCM as a tool to gather the constructs and variables of this study into a common framework.

Schwartz (1994) defined values as “desirable transsituational goals, varying in importance, that serve as guiding principles in the life of a person or other social entity” (p. 21). Personal values express what is important to people in their lives (Bardi and Schwartz 2003); they motivate people's actions and are associated with perception, cognition and behavior (Nazirova and Borbala 2024; Sagiv and Schwartz 2022). Therefore, values may also motivate CAIM use. Schwartz's (1992) theory differentiates between 10 basic values that form a motivational continuum according to the similarities (compatibilities) and differences (conflicts) of their motivating goals. These values can be combined into broader higher‐order values (Davidov 2008; Lechner et al. 2024) or further divided (Schwartz 2012). We take the former approach and focus on four higher‐order values that form two value dimensions (Schwartz 1992, 1994). The first dimension, conservation versus openness to change (OtC), captures the conflict between conservation values (security, conformity and tradition) emphasizing self‐restriction, preserving traditional practices and protecting stability, and OtC values (self‐direction and stimulation), which prioritize independent thought and action and favor change. People endorsing conservation (vs. OtC) may be less likely to use CAIM because they are motivated to pursue the stability, order and protection that established health care authorities can provide rather than to try new or unconventional modalities (Ahola 2020). Indeed, valuing conservation (vs. OtC) has been found to be negatively associated with past CAIM use and with the intention to use CAIM—the latter of which was measured as a preference to seek advice or treatment from a CAIM practitioner in hypothetical symptom scenarios (Ahola 2020).

The second dimension, self‐transcendence versus self‐enhancement, captures the conflict between self‐transcendence values (universalism and benevolence), emphasizing concern for the welfare of others, and self‐enhancement values (power and achievement), which prioritize one's own relative success and dominance over others. Hedonism is located between OtC and self‐enhancement values in the value structure. Conceptually, the motivations underlying the self‐transcendence versus self‐enhancement value dimension do not seem as relevant in the context of CAIM use compared with the conservation versus OtC dimension. However, since the self‐transcendence versus self‐enhancement value dimension is positively associated with past CAIM use (Ahola 2020), and because considering the full spectrum of values is needed for a thorough understanding of values‐behavior relationships (Sagiv and Roccas 2021; Sagiv et al. 2017), we examine both value dimensions in relation to CAIM use.

### 1.2 Health Beliefs

While values vary in how important different goals are considered as guiding principles in one's life, beliefs vary in the degree to which individuals consider certain propositions about relationships to be true (Schwartz 2012). Approaches to health, healing, and the treatment of illness have been discussed from two seemingly opposing perspectives: holistic and reductionistic. Holism has been conceptually linked with CAIM, and holistic health beliefs have been shown to be empirically associated with CAIM use (Bishop et al. 2007; Kelner and Wellman 1997; Siahpush 1999). Reductionism, in turn, is typically considered a characteristic of conventional medicine (Graham et al. 2021; Hoenders et al. 2024; Rocca and Anjum 2020), even though reductionistic and holistic approaches are intertwined in science (Mazzocchi 2012) and in health‐care practices (Beresford 2010; Vogt et al. 2016).

Holism refers to any doctrine emphasizing the priority of a whole over its parts (Blackburn 2016)—a mode of thinking that originates from Aristotelian philosophy (Beresford 2010; Politis 2004). Holistic medicine, in turn, views patients as “mental and emotional, social and spiritual, as well as biological and physiological beings” (Gordon 1990, 358). CAIM use has been viewed as “irregular,” “unconventional” and “alternative”, falling outside conventional health care (Bicho et al. 2023; Eisenberg et al. 1993; Gale 2014; Sirois et al. 2016) and has been claimed to reflect a belief in an “alternative treatment ideology”, one aspect of which is a holistic understanding of health (Kelner and Wellman 1997). Holistic views contend that value‐laden phenomena (e.g., vital goals, meaning and purpose) play a central role in health and healing (Saad and Prochaska 2020).

A holistic understanding of health as involving an integration of mind, body, and spirit (Siahpush 1999) is referred to as holistic health beliefs (Hyland et al. 2003). Studies have reported a positive relationship between holistic health beliefs and CAIM use (e.g., Bryden et al. 2018; Sharif et al. 2020) although not uniformly (for a review, see Bishop et al. 2007). In addition, a holistic approach to healing is associated with the clinical work of CAIM practitioners (Graham et al. 2021, 2023).

Reductionism refers to a scientific paradigm that can be traced back to Descartes's materialism (Greene and Loscalzo 2017), arguing that deconstructing a complex process into its component parts enables better comprehension (Beresford 2010; Rocca and Anjum 2020, 77). In Western societies, a naturalistic philosophy of health, referred to as reductionistic health beliefs, is associated with conventional medicine (Graham et al. 2021; Rocca and Anjum 2020, 78). This approach represents the default health care model (Hoenders et al. 2024) that has historically considered health as the absence of disease (Boorse 1977). Reductionistic health beliefs reflect ontological reductionism/naturalism related to conventional medicine (Beresford 2010; Politis 2004), which considers reduction from tissues to cells and further to molecules as key to explaining the mechanism of a cure. Reductionistic and naturalistic views provide insight into physical, natural, biological or physiological processes that are tangible (in the material sense), observable and measurable using modern technology (Saad and Prochaska 2020).

Based on the conceptual and empirical connections between holistic health beliefs and CAIM use, we expect a positive association between holistic health beliefs and the past and intended future use of CAIM. Reductionistic health beliefs have not previously been studied in relation to CAIM use. Since these beliefs contrast with holistic health beliefs, we expected an inverse relationship.

Furthermore, as holistic beliefs are seen to characterize CAIM users (Bishop et al. 2007; Kelner and Wellman 1997; Siahpush 1999), we assume that CAIM users will endorse holistic health beliefs more than reductionistic health beliefs. Regarding non‐users of CAIM, we make no assumptions with regard to the level of holistic compared with reductionistic health beliefs.

### 1.3 Experienced Benefits and Harms of CAIM Use

While a considerable amount of research on socioeconomic factors has explained CAIM use (Fjær et al. 2020; Guillaud et al. 2019), there is limited research on the experienced benefits and harms related to it. In the existing literature, the perceived benefits of CAIM use include the management of pain (Bhoi et al. 2021) and perceived improvement of physical and emotional health, well‐being and quality of life (Maghrabi et al. 2025; Tangkiatkumjai et al. 2020; Wode et al. 2019). From a practical standpoint, CAIM has been reported by users as helpful in multiple cross‐sectional surveys (Kristoffersen et al. 2021; Pyykkönen et al. 2023). The existing evidence suggests that the user's subjective experience plays a central role in how CAIM use is evaluated and highlights the importance of examining the role of perceived benefits and harms in understanding the continued use of CAIM. Based on earlier research showing that perceived and expected benefits are associated with current CAIM use (Tangkiatkumjai et al. 2020), we assume that having experienced benefits is linked to an intention to continue use.

Research on the harms related to CAIM use remains limited (Ernst 2022). Some studies reporting possible harms of CAIM use have considered them within the context of adverse effects following concomitant use of natural remedies and prescription drugs—that is, herb–drug interactions (Ekor 2014; Posadzki et al. 2013). Other harms reported by CAIM users include temporary discomfort or pain and the high cost of CAIM treatments (Mora et al. 2022; Oh et al. 2010; Wode et al. 2019).

Risks associated with CAIM use can be conceptualized as either direct risks or indirect risks (Stub et al. 2016). Direct risks refer to harms that arise directly from specific products or from CAIM interventions, such as consumption of toxic products, adverse reactions, or other physical harms resulting. In contrast, indirect risks relate to the broader context of the CAIM treatments used, including the care provider setting and communication between the provider and the CAIM user. Although indirect risks do not stem directly from CAIM modalities, their use can have significant implications for patient safety—for example, by delaying relevant conventional treatment. Both indirect and direct risks may therefore pose a significant threat to CAIM users' safety (Stub et al. 2016), and this conceptualisation of risks has been previously applied in the context of CAIM use in cancer care (Stub et al. 2018). Since there is a lack of research on the associations between experienced harms and current or future intended CAIM use, the extent to which experiencing harms from CAIM use relates to the intention to continue use in the future remains unclear. We explore this relationship in this study.

### 1.4 Rationale for the Study

To summarize, the rationale for this study is twofold: to examine the factors associated with future intention to use CAIM that have rarely been examined, and to include personal values, health beliefs, and experiences of CAIM use as person‐centred factors that have been shown to be associated with CAIM use but have not been studied jointly in this context. This is the first study to empirically examine reductionistic health beliefs and relate them to holistic health beliefs and CAIM use. Specific hypotheses and research questions are shown in Table 1, along with the key concepts of the study.

**TABLE 1:** Concepts, their dimensions and hypotheses.

Concept	Dimensions/aspects	Hypotheses
Personal values (Schwartz 1992)	Conservation versus openness to change	H1. Low levels of conservation (vs. OtC) values are positively associated with (a) past CAIM use and (b) the intention to use CAIM in the future.
	Self‐transcendence versus self‐enhancement	H2. High levels of self‐transcendence (vs. self‐enhancement) values are positively associated with (a) past CAIM use and (b) the intention to use CAIM in the future.
Health beliefs (e.g., Bishop et al. 2005; Hyland et al. 2003)	Holistic health beliefs	H3. High levels of holistic health beliefs are positively associated with (a) past CAIM use and (b) the intention to use CAIM in the future.
	Reductionistic health beliefs	H4. Low levels of reductionistic health beliefs are positively associated with (a) past CAIM use and (b) the intention to use CAIM in the future.
	Endorsement of holistic versus reductionistic health beliefs	H5. CAIM users endorse holistic health beliefs more than reductionistic health beliefs. RQ1. To what extent do non‐users of CAIM endorse holistic versus reductionistic health beliefs?
Experiences of CAIM use (Tangkiatkumjai et al. 2020)	Experienced benefits of CAIM use	H6. CAIM users who report having experienced benefits from CAIM use are more likely to intend to continue CAIM use in the future than CAIM users who have not experienced benefits.
	Experienced harms of CAIM use	RQ2. Is having experienced harms from CAIM use (vs. no experienced harms) related to the intention to continue CAIM use?
		H7. (a) Low levels of conservation (vs. OtC) values, (b) high levels of self‐transcendence (vs. self‐enhancement) values, (c) high levels of holistic health beliefs and (d) low levels of reductionistic health beliefs are positively associated with the intention to continue CAIM use.

## 2 Methods

### 2.1 Participants

Data were collected in December 2022 in Finland from a commercial probability‐based online panel provided by Kantar Public (currently Verian Group) using an online questionnaire. This panel comprises approximately 30,000 people aged 15 years and above recruited as panelists to represent the Finnish population. Invitations to participate in this study were sent to 11,507 randomly selected panel members aged 16 years and above living in mainland Finland. We obtained 3244 responses, with a response rate of 28.2%. The demographic characteristics are shown in Table 2. This study is part of a larger Finnish CAIM research project, and the survey included questions in addition to those reported here (for more details on the data collection procedure, see Pyykkönen et al. 2023). Each respondent provided informed consent to participate in the study. The data were weighted by age, gender and region of residency to increase their representativeness. IBM SPSS Statistics (version 29) was used to analyze the data, and the weight measure was used in all subsequent analyses.

**TABLE 2:** Demographic characteristics of the study participants (*N* = 3244).

Variable	% unweighted	% weighted
Gender		
Female	52.0	50.8
Male	48.0	49.2
Age group		
16–29 years	12.0	17.0
30–59 years	46.1	47.1
≥ 60 years	41.9	35.9
Household income		
≤ €35,000	26.6	27.9
€35,001–€50,000	17.4	17.1
≥ €50,001	41.2	40.2
Response missing	14.7	14.8
Level of education		
Primary and lower secondary school	8.4	8.7
Upper secondary or vocational college education	48.8	48.8
Bachelor's degree or higher vocational degree	23.2	23.3
Master's degree or higher	19.1	18.5
Response missing	0.6	0.7

### 2.2 Measures

#### 2.2.1 Dependent Variables

##### 2.2.1.1 CAIM Use

Operational definition: The term CAIM was considered to encompass health systems, modalities, products and practices not typically considered part of conventional medicine that are used to prevent or treat illness or to promote health and well‐being (Lee et al. 2022; Zollman and Vickers 1999) and that are considered CAIM in Finland (Pyykkönen et al. 2023), including the following: (a) *CAIM providers and therapists*: chiropractors, homeopaths, acupuncturists, phytotherapists, bone setters, energy healers, reflexologists, massage therapists (other, non‐conventional), naprapaths, osteopaths, art therapists, cuppists, hypnotherapists, traditional Chinese medicine (TCM) practitioners, anthroposophic therapists and ayurvedic practitioners; (b) *CAIM products* (natural remedies): herbs and herbal medicines, homeopathic remedies and other supplements (excluding vitamins and minerals); and (c) *CAIM self‐help practices*: meditation and mindfulness, yoga, tai chi and qigong, relaxation techniques, visualization, attending traditional healing ceremonies, and neurolinguistic programming (NLP).

CAIM use was measured using a modified Finnish version of the International Questionnaire to Measure Use of Complementary and Alternative Medicine (I‐CAM‐QFI; Pyykkönen et al. 2023) which is based on the original English I‐CAM‐Q (Quandt et al. 2009) and its modified Norwegian version (Kristoffersen et al. 2021). The questionnaire was translated from Norwegian and English into Finnish using a back‐translation procedure. The lists of CAIM providers and self‐help practices were adapted to fit the Finnish context (for details, see Pyykkönen et al. 2023).

The respondents were presented with lists of providers, products and self‐help practices and were asked to indicate which they had visited or used during the previous 12 months. A dichotomous variable was computed differentiating *CAIM users* (1 = visited at least one CAIM provider, used at least one CAIM product type, or used at least one CAIM self‐help practice) from *non‐CAIM users* (0 = did not use any of these) according to the CAIM operational definition, excluding providers (physicians and conventional massage therapists), products (vitamins and minerals) and self‐help practices (e.g., sauna and prayer) that do not fall within the scope of CAIM. Regarding visiting CAIM providers, 22 (out of 199) respondents who answered the open‐ended question “other, what” were included in the category of CAIM users because their reported practice met the CAIM operational definition (Pyykkönen et al. 2023). Regarding CAIM products and self‐help practices, responses selecting the ‘other’ option were not included in the category of CAIM users. For products, respondents were not asked to specify which additional products they had used; for self‐help practices, the scope of the open‐ended responses (*n* = 130) was too broad (Pyykkönen et al. 2023). Regarding visits to providers and the use of self‐help practices, the presentation order of the modalities was randomized.

##### 2.2.1.2 Intention to Use CAIM/Continue Use

Intention to use CAIM in the future/intention to continue use was measured using a single item developed by the authors: “Do you intend to use any complementary treatments in the future?” This variable was dichotomised to facilitate comparability with the variable measuring CAIM use; the response options “yes” and “probably yes” were combined to reflect the intention to use CAIM in the future (coded as 1), and the response options “no”, “probably not”, and “I do not know/I do not want to answer” were combined to reflect not clear intention (coded as 0). In separate analyses that included only CAIM users, this variable was considered to indicate intention to continue CAIM use.

#### 2.2.2 Independent Variables

##### 2.2.2.1 Personal Values

Personal values were measured using the 10‐item Short Schwartz's Value Survey (SSVS; Lindeman and Verkasalo 2005). The names of 10 basic values—power, achievement, hedonism, stimulation, self‐direction, universalism, benevolence, tradition, conformity, and security—were presented to the participants, accompanied by the related value items from the Schwartz Value Survey (Schwartz 1992), for example: “power (e.g., authority, social power, wealth, control over others).” The participants were asked to indicate the importance of each value using a 9‐point scale (0 = *not important*, 4 = *important*, 8 = *very important*; the response option “don’t know” was also available).^2^ Prior to this, an anchoring procedure was used to address individual differences in scale use; respondents were asked to read through descriptions of each value and choose, first, which value was the most important for them and, second, which value was the least important (Schwartz 1994). The order of the value items was randomized.

The reliability of the overall value scale as estimated by Cronbach's alpha was *α* = 0.73. Two bipolar value dimensions—conservation versus OtC and self‐transcendence vs. self‐enhancement—were computed using equations provided by Taskinen (2018)^3^ that included constants and weights obtained from factor analysis. Since responses to all 10 value items were required to calculate individuals’ scores on these dimensions, we included only individuals who had responded to all these items. Each dimension captured the relative importance of the opposing values for respondents; that is, higher scores on the conservation versus OtC dimension reflected endorsement of conservation more than OtC, and higher scores on the self‐transcendence versus self‐enhancement dimension indicated valuing self‐transcendence more than self‐enhancement.

##### 2.2.2.2 Holistic and Reductionistic Health Beliefs

Existing measures were utilized to formulate the holistic statements and the contrasting reductionistic statements (e.g., Bishop et al. 2005; Lie and Boker 2004; Siahpush 1999). Although items tapping reductionistic health beliefs have previously been included among survey items in some studies (e.g., Bishop et al. 2005), they were used as reversed scores among items measuring holistic beliefs and not considered separately in the analyses. Holistic and reductionistic health beliefs were measured using three items each (see Table 3). One item measured holistic beliefs of health and an opposing item tapped reductionistic beliefs of health, reflecting the definition of health as freedom from disease (Boorse 1977; Hughner and Kleine 2004; Saad and Prochaska 2020). Further, one item measured holistic beliefs about treatment and a contrasting item measured reductionistic beliefs about treatment (cf. Bishop et al. 2005). Finally, one item measured belief in the body's ability to heal itself (i.e., vitalism), which has been associated with CAIM (Graham et al. 2021; McSwan et al. 2021; Ning 2012) and holistic health (Shroff 2011) and has been included in measurement scales for holistic health beliefs (Bishop et al. 2005; Siahpush 1999). A contrasting item tapped disbelief in vitalism, which was considered a reductionistic belief. The respondents were asked to indicate their agreement or disagreement with each statement using a seven‐point Likert scale ranging from 1 (*completely disagree*) to 7 (*completely agree*); the response option “I do not know” was also provided. A mean variable was computed for both holism and reductionism, including respondents who had responded to at least two of the three items measuring each type of beliefs. Cronbach's alpha was 0.60 for holism and 0.49 for reductionism.

**TABLE 3:** Items measuring health beliefs.

Dimension	Holistic health beliefs	Reductionistic health beliefs
Beliefs about health	“Health is the balance (harmony) between body, mind and spirit” (cf. Astin 1998; Bishop et al. 2005, Grzywacz et al. 2012).	“Health is the absence of disease (that there is no disease)” (see Boorse 1977).
Beliefs about treatment	“In healthcare and the treatment of illness, one should focus on the whole person” (cf. Bishop et al. 2005).	“In healthcare and the treatment of illness, one should concentrate only on individual symptoms” (cf. Bishop et al. 2005, Grzywacz et al. 2012).
Belief/disbelief about vitalism	“The body has a natural ability to heal itself” (Bishop et al. 2005; Siahpush 1999).	“The body does not have the ability to heal itself” (Grzywacz et al. 2012).

##### 2.2.2.3 Experienced Benefits of CAIM Use

Experienced benefits of CAIM use were assessed using the following question: “Which of the mentioned benefits have you experienced resulting from using CAIM (visiting providers, using natural remedies and self‐help practices) during the last 12 months?” The answer options were: (1) eased pains, aches, tightness or other ailments; (2) relieved stress, sleeplessness, depression or anxiety; (3) increased mental well‐being, energy or everyday functionality; (4) decreased medication use; (5) I felt heard and seen with my ailments and problems; (6) gave support, faith or hope in solving my problems; and (7) other. Options for no benefits (8) and I do not know (9) were also provided.

##### 2.2.2.4 Experienced Harms of CAIM Use

Experienced harms of CAIM use were assessed using the question: “Which of the mentioned harms have you experienced resulting from using CAIM (visiting providers, using natural remedies and self‐help practices) during the last 12 months?” The answer options were: (1) money was wasted, as I did not get help; (2) I was cheated; (3) pains, aches or ailments became worse; (4) I felt hurt or treated inappropriately; and (5) other. Options for no harms (6) and I do not know (7) were also provided. The response options for benefits and harms were chosen based on earlier research (Aarva et al. 2022; Tangkiatkumjai et al. 2020).

Each respondent could choose all the options that applied, which were treated as *yes* responses. The options that the respondent did not choose were considered *no* responses, along with cases in which the respondent chose the “No benefits” or “No harms” options. For the final analysis, a three‐class variable was created for experienced benefits and harms, respectively: not having experienced any benefits/harms (coded as 0), having experienced at least one of the listed benefits/harms (coded as 1), and ‘I do not know’ (coded as 2).

The open‐ended “other, what?” responses for experienced benefits (*n* = 53) and harms (*n* = 17) were read through by the research team. Most responses were classified as genuine answers (e.g., for benefits: “Maintaining mobility,” for harms: “The ailment eased only for a moment”) and were included in the variables of experienced benefits/harms. Five respondents reported not having used CAIM in the 12 months prior to taking part in the survey, despite belonging to the researcher‐defined CAIM user group. These respondents were placed in the category of non‐CAIM users in the analyses of the total sample.

#### 2.2.3 Control Variables

Demographic variables included *age* (calculated from the respondents' year of birth), *gender* (0 = male, 1 = female), *education* (measured with six categories, combined into four: 1 = primary and lower secondary school, 2 = upper secondary or vocational college education, 3 = bachelor's degree or higher vocational degree, 4 = and master's degree or higher) and *household income* (measured with six categories, combined into three, 1 = ≤€35,000, 2 = €35,001–€50,000, 3 = ≥€50,001). Self‐rated health was assessed by a single question “What is your state of health like at the moment?”—with the response options ranging from 1 (*poor*) to 5 (*good*); the response option “I do not know/I do not want to answer” was coded as missing. Control variables for the statistical analyses were chosen, as they have been shown to be associated with CAIM use (Bishop and Lewith 2010; Fjær et al. 2020; Frass et al. 2012; Guillaud et al. 2019).

### 2.3 Analysis

CCM served as a tool to organize the constructs and variables in a common framework. CCM posits that CAIM use is motivated by its practical (or utilitarian) value to consumers, such as positive physiological and psychological outcomes, as well as its symbolic value, such as a perceived fit between one's own belief system and that of CAIM. We considered the experienced benefits of CAIM use as indicators of its practical value and holistic health beliefs as indicators of its symbolic value. Utilitarian and symbolic values are seen in CCM to contribute to behavioral and psychological commitment to CAIM (Sirois et al. 2016). In this study, past CAIM use represented behavioral commitment to CAIM, and the intention to use CAIM in the future reflected a psychological commitment to CAIM—the latter of which we considered an indicator of intention to continue CAIM use in the case of CAIM users. In addition, we examined the role of reductionistic health beliefs and personal value dimensions (conservation vs. OtC and self‐transcendence vs. self‐enhancement) in behavioral and psychological commitment to CAIM. We also analyzed the role of experienced harms in CAIM users’ intention to continue using CAIM.

The relationships among the study variables were analyzed using Pearson's correlations and binary logistic regression analysis. Health beliefs and personal values were treated as continuous variables in correlation analyses; however, they were treated as categorical—low, average, and high, formed by ranking each variable and distributing the scores to three groups of equal sizes—in logistic regression analyses to facilitate comparability between the effects of variables measured using different scales. In the logistic regression analyses, the effect of each independent variable was first analyzed separately without the control variables. Second, all independent variables were entered simultaneously, and the analyses were adjusted for the effects of the control variables. The analysis of future intended CAIM use was further adjusted for the effects of past CAIM use, since it is associated with the intention to use CAIM (Ben Natan et al. 2016; Furnham and Lovett 2001). Experienced benefits and harms of CAIM use were included in the analyses of CAIM users’ intentions to continue use. As the control variable of household income had many missing responses, the category of missing responses was entered as a separate category in the logistic regression analyses. Endorsement of holistic versus reductionistic health beliefs—treated as continuous variables—was analyzed using paired‐samples *t*‐tests, separately among CAIM users and non‐users. The level of statistical significance was set at *p* < 0.05 in all analyses.

## 3 Results

Descriptive statistics and correlations for the study variables are presented in Table 4. The results of the logistic regression analyses in the total sample, as shown in Table 5, indicated that the conservation (vs. OtC) value dimension was associated with CAIM use; respondents scoring low (vs. high; unadjusted and adjusted analyses) or average (vs. high; only in unadjusted analysis) on conservation values were more likely to have used CAIM. Conservation values were associated with future use intentions only in the unadjusted analysis; respondents scoring low (vs. high) on conservation were more likely to intend to use CAIM in the future. Therefore, as hypothesized, low levels of conservation (vs. OtC) values were positively associated with past CAIM use (H1a), whereas the hypothesis that these values would also be associated with the intention to use CAIM did not receive clear support (H1b). The self‐transcendence (vs. self‐enhancement) value dimension was not associated with past CAIM use. This value dimension was associated with future intention to use CAIM only in the unadjusted analysis; respondents scoring average or high (vs. low) on self‐transcendence were more likely to intend to use CAIM in the future. Thus, the hypothesis that high levels of self‐transcendence (vs. self‐enhancement) would be associated with past CAIM use (H2a) was not supported, and the hypothesized association between these values and the intention to use CAIM in the future (H2b) did not gain clear support.

**TABLE 4:** Descriptive statistics and Pearson's correlations of study variables.

Variable	*n*	*M/%*	SD	1	2	3	4	5	6	7	8	9	10	11	12	13
1. Conservation^a^, ^f^	3123	98.27	10.51	—												
2. Self‐transcendence^b^, ^f^	3123	97.97	10.68	−0.11***	—											
3. Holism^c^, ^f^	3185	5.35	1.02	−0.01	0.25***	—										
4. Reductionism^d^, ^f^	3167	3.31	1.12	−0.005	−0.25***	−0.28***	—									
5. Gender	3244	50.8%^e^	—	−0.14***	0.31***	0.12***	−0.06**	—								
6. Age^f^	3244	50.27	18.73	0.19***	0.26***	0.17***	−0.19***	0.05**	—							
7. Education^f^	3222	—	0.89	−0.16***	−0.02	−0.06**	−0.003	0.04*	0.07***	—						
8. Income^f^	2762	—	0.88	0.03	−0.13***	−0.06**	−0.05**	−0.11***	0.03	0.34***	—					
9. Self‐rated health^f^	3206	3.54	0.99	−0.03	−0.06**	0.07***	−0.02	−0.01	−0.06***	0.15***	0.20***	—				
10. Has used CAIM	3244	50.9%^g^	—	−0.13***	0.02	0.08***	−0.04*	0.16***	−0.22***	0.04*	−0.03	−0.02	—			
11. Intends to use CAIM^h^	3244	42.0%^g^	—	−0.10***	0.11***	0.23***	−0.14***	0.19***	−0.03	0.02	0.01	0.005	0.35***	—		
12. CAIM benefits^h^, ^i^	1652	79.9%^g^	—	−0.09***	0.006	0.06*	−0.01	0.09***	−0.05	0.03	0.04	−0.02	—	0.43***	—	
13. CAIM harms^h^, ^i^	1652	15.0%^g^	—	0.13***	−0.33***	−0.27***	0.25***	−0.25***	−0.25***	−0.06*	0.04	−0.06*	—	−0.18***	0.06*	—

*Note:* Two‐tailed tests. Analyses were based on weighted data.

^a^ Conservation versus openness to change value dimension.

^b^ Self‐transcendence versus self‐enhancement value dimension.

^c^ Holistic health beliefs.

^d^ Reductionistic health beliefs.

^e^ % female.

^f^ Variable treated as continuous in this table.

^g^ % yes.

^h^ Categories “no” and “I do not know” combined.

^i^ CAIM users only.

* *p* < 0.05.

** *p* < 0.01.

*** *p* < 0.001.

**TABLE 5:** Logistic regressions of past and intended future use of CAIM in the total sample.

Variable	Has used CAIM					
	1a. Unadjusted analyses			1b. Adjusted analysis		
	OR	95% CI	*p*	OR	95% CI	*p*
Conservation^a^			< 0.001			0.001
Low	1.85	1.55; 2.20		1.43	1.18; 1.73	
Average	1.29	1.09; 1.54		1.13	0.94; 1.36	
High	1.00			1.00		
Self‐transcendence^b^			0.270			0.707
Low	1.00			1.00		
Average	0.93	0.79; 1.11		0.92	0.76; 1.12	
High	1.08	0.91; 1.28		0.96	0.78; 1.18	
Holism^c^			< 0.001			< 0.001
Low	1.00			1.00		
Average	1.10	0.92; 1.31		1.10	0.90; 1.33	
High	1.55	1.29; 1.85		1.75	1.42; 2.16	
Reductionism^d^			0.009			0.020
Low	1.28	1.08; 1.52		1.32	1.08; 1.61	
Average	1.03	0.87; 1.22		1.07	0.89; 1.29	
High	1.00			1.00		

	Intends to use CAIM					
	2a. Unadjusted analyses			2b. Adjusted analysis		
Conservation^a^			< 0.001			0.077
Low	1.60	1.35; 1.91		1.23	1.00; 1.51	
Average	1.11	0.93; 1.32		1.01	0.83; 1.23	
High	1.00			1.00		
Self‐transcendence^b^			< 0.001			0.659
Low	1.00			1.00		
Average	1.35	1.13; 1.61		1.06	0.87; 1.31	
High	1.68	1.41; 2.01		1.11	0.89; 1.37	
Holism^c^			< 0.001			< 0.001
Low	1.00			1.00		
Average	2.19	1.82; 2.65		2.03	1.65; 2.51	
High	3.42	2.82; 4.14		2.74	2.19; 3.42	
Reductionism^d^			< 0.001			< 0.001
Low	2.07	1.74; 2.48		1.55	1.25; 1.91	
Average	1.36	1.14; 1.62		1.20	0.99; 1.47	
High	1.00			1.00		
Has used CAIM^e^			< 0.001			< 0.001
No	1.00			1.00		
Yes	4.35	3.74; 5.05		3.88	3.28; 4.58	

*Note:* Analyses were based on weighted data. Unadjusted analyses 1a and 2a: All variables were analyzed separately; *n* = 3123–3185. Adjusted analyses 1b and 2b included the variables *holism*, *reductionism*, *conservation* and *self‐transcendence* and were adjusted for control variables; *n* = 3051–3052. Analysis 2b also included the variable *has used CAIM*.

^a^ Conservation versus openness to change value dimension.

^b^ Self‐transcendence versus self‐enhancement value dimension.

^c^ Holistic health beliefs.

^d^ Reductionistic health beliefs.

^e^ Used at least one type of CAIM at least once during the previous 12 months.

Both holistic and reductionistic health beliefs were associated with the past and intended future use of CAIM. Respondents scoring high (vs. low) on holistic health beliefs were more likely to have used CAIM, and those scoring average or high (vs. low) on holism were more likely to intend to use CAIM in the future. In both unadjusted and adjusted analyses, the results support the hypothesis that high levels of holistic health beliefs would be positively associated with both past and intended future CAIM use (H3a and H3b). Respondents scoring low (vs. high) on reductionistic health beliefs were more likely to have used CAIM and to intend to use it in the future (unadjusted and adjusted analyses). In addition, in the unadjusted analysis, respondents scoring average (vs. high) on reductionism were more likely to intend to use CAIM. These results support the hypothesis that low levels of reductionistic health beliefs would be positively associated with both past and intended future CAIM use (H4a and H4b). Having used CAIM previously showed the strongest association with the intention to use CAIM in the future.

Paired‐samples *t*‐tests comparing the levels of holistic versus reductionistic health beliefs indicated that CAIM users scored higher on holism (*M* = 5.43; SD = 1.03) than on reductionism (*M* = 3.27; SD = 1.15; *t*[1624] = 49.73, *p* < 0.001), supporting the assumption that CAIM users would endorse holistic health beliefs more than reductionistic health beliefs (H5). Regarding research question 1, which asks “to what extent do non‐users of CAIM endorse holistic versus reductionistic health beliefs?”, the results showed that non‐users of CAIM also scored higher on holism (*M* = 5.26; SD = 1.02) than on reductionism (*M* = 3.36; SD = 1.08; *t*[1536] = 45.12, *p* < 0.001).

In the CAIM user group (*n* = 1652), 58.8% of the respondents intended to use CAIM in the future, 26.6% did not intend to do so, and 14.6% did not know. Logistic regression analyses (Table 6) showed that both experienced benefits and harms of CAIM use were related to the intention to continue CAIM use in the future (unadjusted and adjusted analyses). Having experienced at least one type of benefit from CAIM use (compared with not having experienced benefits) and not having experienced any harms (compared with having experienced at least one type of harm) were associated with an increased likelihood of intending to continue CAIM use. These results support the hypothesis that CAIM users who report having experienced benefits from CAIM use would be more likely to intend to continue compared with CAIM users who have not experienced benefits (H6). They also answer research question 2, which asks whether having experienced harms from CAIM use (vs. no experienced harms) is related to the intention to continue CAIM use.

**TABLE 6:** Logistic regressions of CAIM users' intention to continue CAIM use.

Variable	Intends to continue CAIM use					
	3a. Unadjusted analyses			3b. Adjusted analysis		
	OR	95% CI	*p*	OR	95% CI	*p*
Conservation^a^			< 0.001			0.180
Low	1.64	1.29; 2.10		1.34	0.98; 1.82	
Average	1.25	0.97; 1.61		1.16	0.85; 1.57	
High	1.00			1.00		
Self‐transcendence^b^			< 0.001			0.675
Low	1.00			1.00		
Average	1.53	1.20; 1.95		0.97	0.70; 1.33	
High	1.96	1.53; 2.50		1.11	0.79; 1.55	
Holism^c^			< 0.001			< 0.001
Low	1.00			1.00		
Average	2.26	1.75; 2.93		2.02	1.48; 2.75	
High	3.54	2.73; 4.60		2.59	1.87; 3.60	
Reductionism^d^			< 0.001			0.015
Low	2.25	1.76; 2.88		1.60	1.16; 2.20	
Average	1.56	1.22; 1.99		1.32	0.98; 1.77	
High	1.00			1.00		
CAIM benefits^e^			< 0.001			< 0.001
No	1.00			1.00		
Yes	19.33	11.05; 33.80		17.10	9.50; 30.75	
Don't know	2.31	1.21; 4.41		2.43	1.21; 4.86	
CAIM harms^f^			< 0.001			< 0.001
No	3.76	2.83; 5.00		2.17	1.51; 3.11	
Yes	1.00			1.00		
Don’t know	0.53	0.36; 0.79		0.87	0.52; 1.46	

*Note:* Analyses were based on weighted data. Unadjusted analyses 3a: All variables were analyzed separately; *n* = 1611–1652. Adjusted analysis 3b included the variables *holism*, *reductionism*, *conservation*, *self‐transcendence*, *CAIM benefits* and *CAIM harms* and was adjusted for control variables; *n* = 1575.

^a^ Conservation versus openness to change value dimension.

^b^ Self‐transcendence versus self‐enhancement value dimension.

^c^ Holistic health beliefs.

^d^ Reductionistic health beliefs.

^e^ Experienced benefits from CAIM use.

^f^ Experienced harms from CAIM use.

The conservation and self‐transcendence value dimensions were related to the intention to continue CAIM use, but only in unadjusted analyses. CAIM users who scored low (vs. high) on conservation values and those who scored high (vs. low) on self‐transcendence values were more likely to intend to continue CAIM use. Since these associations were statistically nonsignificant in the adjusted analyses, the hypothesis proposing that low levels of conservation (vs. OtC) values and high levels of self‐transcendence (vs. self‐enhancement) values would be positively associated with the intention to continue CAIM use (H7a and H7b) was not clearly supported. Both holistic and reductionistic health beliefs were related to the intention to continue CAIM use. Respondents scoring high or average (vs. low) on holistic beliefs or low (vs. high) on reductionistic beliefs were more likely to intend to continue CAIM use (unadjusted and adjusted analyses). In addition, those scoring average (vs. high) on reductionistic beliefs were more likely to intend to continue CAIM use (only in unadjusted analysis). The findings support the assumption that high levels of holistic health beliefs and low levels of reductionistic health beliefs are positively associated with the intention to continue CAIM use (H7c and H7d).^4^

## 4 Discussion

This study examined the relationships of personal values and health beliefs with CAIM use and use intentions among Finnish people. It also investigated the extent to which CAIM users and non‐users endorse holistic versus reductionistic health beliefs. As expected, the results showed that individuals who placed less importance on conservation (vs. OtC) values were more likely to have used CAIM, whereas the self‐transcendence (vs. self‐enhancement) value dimension was not associated with prior CAIM use. Neither of these two value dimensions was clearly linked with the intention to use CAIM in the future nor with CAIM users' intention to continue use. The results indicate that the conservation versus OtC value dimension plays a clearer role in CAIM use than the value dimension of self‐transcendence versus self‐enhancement, in accordance with earlier findings (Ahola 2020). This can be understood when considering the motivating goals of values. OtC values involve seeking new and stimulating ideas and trying new things, which also characterize openness‐to‐experience personality traits (Fischer and Boer 2015) that have been empirically linked to CAIM use (for a review, see Galbraith et al. 2018). Therefore, CAIM may offer users something new or bring about a practical change compared with the methods used previously (or concurrently) for health promotion or illness treatment.

Regarding health beliefs, past and intended future CAIM use as well as CAIM users' intention to continue use were linked with higher endorsement of holistic health beliefs and lower endorsement of reductionistic health beliefs. One reason for the appeal of CAIM for those endorsing holistic health beliefs may be that since the holistic approach is linked with the clinical work of CAIM practitioners (Graham et al. 2021, 2023), CAIM may provide users with holistic care experiences.^5^ In contrast, those endorsing reductionistic health beliefs may find the philosophical foundation of CAIM, based on holism and vitalism (Graham et al. 2021), incompatible with their beliefs regarding health and the treatment of illness. Our findings contribute to earlier studies demonstrating a positive relationship between holistic health beliefs and CAIM use (for a review, see Bishop et al. 2007) by showing that these beliefs are also linked to use intentions. Reductionistic health beliefs had not previously been analyzed in relation to CAIM use; thus, our findings provide novel insights into the role of these beliefs in CAIM use and use intentions.

In addition to being the first study to empirically examine reductionistic health beliefs, this is the first to compare the extent to which people endorse holistic versus reductionistic health beliefs. Both CAIM users and non‐users scored higher on holistic than on reductionistic health beliefs, which indicates that the Finnish public, in general, subscribes more to a holistic than a reductionistic understanding of health and the treatment of illness. This novel finding shows that a holistic approach is important for people regardless of whether they use CAIM. This indicates that people view health as a broad biopsychosocial phenomenon. This is in line with the WHO conception of health declared in its constitutional document in 1948 (WHO 2020, 1–19) and is also considered crucial in healthcare, particularly in general practice (Thomas et al. 2020).

Whereas most existing research on CAIM use has examined past use, this study incorporated a future temporal perspective by examining CAIM use intentions. Prior CAIM use showed the strongest link with future use intention. This is in line with the finding that past CAIM use is associated with the intention to use CAIM (Ben Natan et al. 2016; Furnham and Lovett 2001). Furthermore, having experienced benefits from CAIM use was the factor most strongly associated with CAIM users' intention to continue use. These results suggest that the intention to use CAIM is related to the experienced everyday effectiveness of the CAIM modalities employed; that is, CAIM is used when it is perceived as useful. This is in line with Maghrabi et al.'s (2025) conclusion that individuals' CAIM use is primarily motivated by the perceived usefulness of a particular CAIM modality in addressing their specific needs or goals.

### 4.1 Implications

Consistent with the assumptions of the CCM framework (Sirois et al. 2016), the results of this study indicate that holistic health beliefs are compatible with psychological (i.e., intentions) and behavioral (i.e., use) commitment to CAIM. Furthermore, reductionistic health beliefs and conservation values appear to be incompatible with CAIM commitment. While health beliefs were associated with both types of CAIM commitment, after adjusting for the effects of the control variables, personal values were related only to behavioral CAIM (non‐)commitment. Therefore, compared with health beliefs, the role of personal values in understanding CAIM commitment seems less prominent. This may reflect that values are more abstract and conceptually more distant from CAIM commitment than health beliefs. Our finding that health beliefs contributed to the intention to use CAIM and to continue use, even after adjusting for the effects of previous CAIM use experiences (i.e., having used CAIM and having experienced benefits or harms from CAIM use), further highlights the relevance of health beliefs in the context of CAIM use intentions. Previous studies have found the sizes of the associations between values and CAIM use to be small (Abheiden et al. 2020; Ahola 2020; Messerli‐Rohrbach 2000), which led Abheiden et al. (2020) to conclude that personal values “seem to be a negligible motivation for the use and approval of CAM.” (p. 10). Our findings indicate that some values indeed motivate CAIM use, whereas health beliefs and prior experiences of CAIM seem more useful, particularly in understanding CAIM use intentions. Nevertheless, the results clearly indicate that experienced effectiveness, or perceived usefulness (Maghrabi et al. 2025), plays a more central role than beliefs or values when people make choices regarding CAIM use. This indicates that users' justification for use and intention to use are based on experiences, pragmatically considering usefulness as everyday effectiveness, which is understandable since health itself is a value that many people consider worth pursuing (Aavik and Dobewall 2017).

Even though we measured holistic and reductionistic health beliefs as separate factors and discussed them as opposing entities in the present study, it is important to note that reductionism and holism are not opposing philosophies: “they work best in tandem” (Wuppuluri and Stewart 2020, p. xii). Indeed, whole‐person care (i.e., holistic or biopsychosocial care) is considered a cornerstone of general practice (Thomas et al. 2020).

We consider it important to make a distinction between reductionism in conventionally orientated clinical research and reductionism in health‐care practices. Although the basic assumptions of health or treatment mechanisms in conventional medicine may be seen as reductionistic, this does not mean that health care in practice—that is, all health services available to the population—would be reductionistic or that service users would be proponents of a reductionistic perception of health (for a recent discussion on holism vs. reductionism, see e.g., Buzzoni et al. 2022; Hansson 2022; Hoenders et al. 2024; Jütte 2024; Rocca and Anjum 2020; Saad and Prochaska 2020). Given individuals' various needs, in many cases a reductionistic approach is essential, and in others this could be complemented with a holistic approach.

### 4.2 Limitations

This study is not without limitations. First, the data are not fully representative of the Finnish population. However, using data drawn from a probability‐based online panel and using data weights in the analyses increased the generalisability of the findings. Second, the study's cross‐sectional design did not allow causal conclusions from personal values and health beliefs to CAIM use. It is possible that CAIM use affects health beliefs by reinforcing holistic health beliefs and weakening reductionistic health beliefs, thus affecting future CAIM use intentions. Therefore, longitudinal designs are needed not only to gain further understanding of these relationships but also to identify the extent to which the intention to use CAIM actually predicts future use.

The third limitation relates to the measurement instruments. Holistic and reductionistic health beliefs were measured using only three items each, which may have contributed to obtaining suboptimal levels of reliability, as measured with Cronbach's alpha. In prior studies (e.g., Bishop et al. 2005; Lie and Boker 2004; Siahpush 1999), different measurement instruments have been used to capture holistic health beliefs, rather than having a more widely used, established instrument. This study is the first to attempt to measure reductionistic health beliefs. Regarding measuring personal values, due to space limitations in the questionnaire, we used a 10‐item measurement instrument (SSVS; Lindeman and Verkasalo 2005), whereas using a more extensive instrument, such as the Schwartz Value Survey (SVS, Schwartz 1992) or the Revised Portrait Value Questionnaire (PVQ‐RR, Schwartz 2017), could have provided a more nuanced picture of the studied associations. Similarly, to decrease the time burden of the full questionnaire, the experienced benefits and harms were assessed for general CAIM use instead of for each individual CAIM modality. Furthermore, the questionnaire did not specify which modalities are integrated into the Finnish conventional healthcare system, nor did we ask the respondents which modalities they considered as integrated or which ones they had used as complementary or alternative to conventional healthcare. Finally, recall bias may have influenced respondents’ answers regarding the CAIM modalities they had used (Jensen and Ekholm 2021) or the benefits and harms they had experienced.

### 4.3 Future Research Directions

It has been suggested that holistic medicine has been “somewhat hijacked by alternative therapists” and that it is mistakenly thought to conflict with conventional medicine (Beresford 2010, 722). Researchers have emphasized that CAIM is not holistic if used in isolation (Thomas et al. 2018). Hence, Saba and Tagliagambe (2023) suggested that because reductionism has limitations, such as losing sight of the patient as a whole person, a truly comprehensive understanding of human health will require a synthesis of reductionist and holistic approaches (see also Hoenders et al. 2024). It would be useful to empirically scrutinize how patients and healthcare professionals see this integration and what such a synthesis would look like in health services. Future studies could further develop measurement instruments to gauge synthesized and integrated health beliefs and to incorporate qualitative methods. This might open new possibilities for discussion of health beliefs and reduce the need to oppose holistic and reductionistic viewpoints.

Although not assessed in this study, harms relating to CAIM use can be separated into direct and indirect risks (Stub et al. 2016, 2018). In our analysis, the included experienced harms were grouped together; thus, the final variable included mixed response options that could have been conceptualized as direct (e.g., the statement “pains, aches or ailments became worse”) and indirect risks (e.g., the statement “money was wasted, as I did not get help”). For future studies, it would be imperative to investigate the relationship between experienced harms and the intention to continue CAIM use, while explicitly distinguishing between direct and indirect harms. Such differentiation would provide a more nuanced understanding of how different types of negative experiences, including direct and indirect harms, influence CAIM users' intention to continue or not to continue use.

## 5 Conclusion

This study contributes to the understanding of CAIM use and people's perceptions of health and treatment of illness. We replicated the findings of earlier studies in that individuals endorsing holistic health beliefs are more likely to have used CAIM and that those valuing conservation (over OtC) are less likely to have done so. The novelty of this study lies in empirically examining reductionistic health beliefs for the first time—showing that they are inversely related to CAIM use and use intentions—and contrasting them with holistic health beliefs. The study showed that holistic health beliefs cannot be simply equated with CAIM, since both CAIM users and non‐users were found to believe more in a holistic than in a reductionistic view of health and the treatment of illness. The finding that prior experiences with CAIM most strongly relate to use intentions demonstrates the importance of the experienced everyday effectiveness of CAIM.

## Author Contributions

**Salla Ahola:** conceptualization; data curation; formal analysis; funding acquisition; investigation; methodology; writing – original draft; writing – review and editing. **Maija Pyykkönen:** conceptualization; data curation; funding acquisition; investigation; writing – original draft; writing – review and editing. **Pauliina Aarva:** conceptualization; data curation; funding acquisition; investigation; methodology; writing – original draft; writing – review and editing.

## Funding

Child and Nature Foundation, Helsinki, Finland.

## Ethics Statement

The authors declare that this study does not cause any potential risks or harm to survey respondents, and it meets the ethical requirements of research with human participants according to The Finnish National Board on Research Integrity guidelines. Ethical approval was deemed unnecessary by the Academic Ethics Committee of the Tampere region.

## Consent

Each participant provided informed consent to participate in the study.

## Conflicts of Interest

The authors declare no conflicts of interest.

## Data Availability

The data that support the findings of this study are openly available in the Finnish Social Science Data Archive at https://doi.org/10.60686/t‐fsd3958.

## Endnotes

^1^
However, it is noteworthy that the CAIM‐modalities considered part of conventional medicine, as well as the terminology and discourses used to describe them, vary across countries and continents.

^2^
In contrast to Lindeman and Verkasalo (2005), we did not include the response option 0 = *opposed to my principles*, as its inclusion is important primarily in cross‐cultural studies (Schwartz 1994). Furthermore, following Taskinen (2018), we used a slightly adapted list of the related value items for universalism and benevolence.

^3^
The method was originally used by Lindeman and Verkasalo (2005), and the equations were updated by Taskinen (2018).

^4^
Additional logistic regression analyses were conducted to further evaluate the robustness of the associations between independent and dependent variables. Analyses in which the “I do not know/I do not want to answer” responses were excluded from the variables of CAIM use, use intentions and experienced benefits and harms yielded no substantive changes to the results. In an analysis where values and health beliefs were treated as continuous variables, the association between the conservation value dimension and the intention to use CAIM was statistically significant (*p* = 0.035, OR = 0.99, 95% CI = 0.98; 0.999) in the adjusted analysis, and there were no other substantive changes to the results.

^5^
However, it is worth noting that the levels of holism may vary between different CAIM modalities (Ribeaux and Spence 2001).

## References

[sjop70104-bib-0001] Aarva, P. , T. Väänänen , and T.‐T. Kankkunen . 2022. “Elämäni on Muuttunut Kokonaan: Tutkimus Perusteluista Luontaishoitojen käytölle Ja niistä Saadulle Avulle [My Life's Been Transformed: A Study of the Motivations for CAM Use and the Accounts of the Perceived Benefits of CAM].” Elore 29, no. 2: 103–128. 10.30666/elore.120898.

[sjop70104-bib-0002] Aavik, T. , A. Aavik , and M. Punab . 2014. “Personal Values That Support and Counteract Utilization of a Screening Test for Prostate Cancer.” Behavioral Medicine 40: 22–28. 10.1080/08964289.2013.831805. 24512362

[sjop70104-bib-0003] Aavik, T. , and H. Dobewall . 2017. “Where Is the Location of “Health” in the Human Values System? Evidence From Estonia.” Social Indicators Research 131, no. 3: 1145–1162. 10.1007/s11205-016-1287-4.

[sjop70104-bib-0004] Abheiden, H. , M. Teut , and A. Berghöfer . 2020. “Predictors of the Use and Approval of CAM: Results From the German General Social Survey (ALLBUS).” BMC Complementary Medicine and Therapies 20: 183. 10.1186/s12906-020-02966-9. PMC7291752 32527256

[sjop70104-bib-0005] Ahola, S. 2020. “Are Personal Values Associated With Preferences in Seeking Health Advice or Treatment in Europe?” Psychology, Health & Medicine 25, no. 3: 282–295. 10.1080/13548506.2019.1659982. 31455086

[sjop70104-bib-0006] Aschemann‐Witzel, J. , and K. G. Grunert . 2015. “Resveratrol Food Supplements: A Survey on the Role of Individual Consumer Characteristics in Predicting the Attitudes and Adoption Intentions of US American and Danish Respondents.” BMC Public Health 15: 110. 10.1186/s12889-015-1348-7. PMC4335419 25885176

[sjop70104-bib-0007] Astin, J. A. 1998. “Why Patients Use Alternative Medicine: Results of a National Study.” Journal of the American Medical Association 279: 1548–1553. 10.1001/jama.279.19.1548. 9605899

[sjop70104-bib-0008] Bardi, A. , and S. H. Schwartz . 2003. “Values and Behavior: Strength and Structure of Relations.” Personality and Social Psychology Bulletin 29, no. 10: 1207–1220. 10.1177/0146167203254602. 15189583

[sjop70104-bib-0009] Ben Natan, M. , M. Perelman , and G. Ben‐Naftali . 2016. “Factors Related to the Intention of Israelis to Use Complementary and Alternative Medicine.” Journal of Holistic Nursing 34, no. 4: 361–368. 10.1177/0898010115619088. 26620812

[sjop70104-bib-0010] Beresford, M. J. 2010. “Medical Reductionism: Lessons From the Great Philosophers.” QJM: Monthly Journal of the Association of Physicians 103, no. 9: 721–724. 10.1093/qjmed/hcq057. 20395240

[sjop70104-bib-0011] Bhoi, D. , D. Jain , R. Garg , et al. 2021. “Complementary and Alternative Modalities (CAM) for Pain Management in Musculoskeletal Diseases (MSDs).” Journal of Clinical Orthopaedics and Trauma 18: 171–180. 10.1016/j.jcot.2021.04.021. PMC8113975 34017678

[sjop70104-bib-0012] Bicho, M. , R. Nikolaeva , and C. Lages . 2023. “Complementary and Alternative Medicine Legitimation Efforts in a Hostile Environment: The Case of Portugal.” Sociology of Health & Illness 45, no. 4: 890–913. 10.1111/1467-9566.13625. 36814085

[sjop70104-bib-0013] Bishop, F. L. , and G. T. Lewith . 2010. “Who Uses CAM? A Narrative Review of Demographic Characteristics and Health Factors Associated With CAM Use.” Evidence‐Based Complementary and Alternative Medicine 7, no. 1: 11–28. 10.1093/ecam/nen023. PMC2816378 18955327

[sjop70104-bib-0014] Bishop, F. L. , L. Yardley , and G. Lewith . 2005. “Developing a Measure of Treatment Beliefs: The Complementary and Alternative Medicine Beliefs Inventory.” Complementary Therapies in Medicine 13, no. 2: 144–149. 10.1016/j.ctim.2005.01.005. 16036173

[sjop70104-bib-0015] Bishop, F. L. , L. Yardley , and G. T. Lewith . 2007. “A Systematic Review of Beliefs Involved in the Use of Complementary and Alternative Medicine.” Journal of Health Psychology 12, no. 6: 851–867. 10.1177/1359105307082447. 17956965

[sjop70104-bib-0016] Blackburn, S. 2016. The Oxford Dictionary of Philosophy. Third ed. Oxford University Press.

[sjop70104-bib-0017] Boorse, C. 1977. “Health as a Theoretical Concept.” Philosophy of Science 44, no. 4: 542–573. 10.1086/288768.

[sjop70104-bib-0018] Brodin Danell, J. A. , R. Danell , and P. Vuolanto . 2020. “Fifty Years of Complementary and Alternative Medicine (CAM) – A Bibliometric Analysis of Publication Activity and General Content of the Publications.” Journal of Scientometric Research 9, no. 3: 268–276. 10.5530/jscires.9.3.34.

[sjop70104-bib-0019] Bryden, G. M. , M. Browne , M. Rockloff , and C. Unsworth . 2018. “Anti‐Vaccination and Pro‐CAM Attitudes Both Reflect Magical Beliefs About Health.” Vaccine 36, no. 9: 1227–1234. 10.1016/j.vaccine.2017.12.068. 29395527

[sjop70104-bib-0020] Buzzoni, M. , L. Tesio , and M. T. Stuart . 2022. “Holism and Reductionism in the Illness/Disease Debate.” In From Electrons to Elephants and Elections. Exploring the Role of Content and Context, edited by S. Wuppuluri and I. Stewart , 743–778. Springer. 10.1007/978-3-030-92192-7_39.

[sjop70104-bib-0021] Davidov, E. 2008. “A Cross‐Country and Cross‐Time Comparison of the Human Values Measurements With the Second Round of the European Social Survey.” Survey Research Methods 2: 33–46. 10.18148/srm/2008.v2i1.365.

[sjop70104-bib-0022] Decree on Health Care Professionals . 1994. “Health Care Professionals Decree No. 564/1994.” In Finnish: Asetus terveydenhuollon ammattihenkilöistä 564/1994. Unofficial English translation. https://www.finlex.fi/fi/lainsaadanto/saadoskaannokset/1994/eng/564.

[sjop70104-bib-0023] Eisenberg, D. M. , R. C. Kessler , C. Foster , F. E. Norlock , D. R. Calkins , and T. L. Delbanco . 1993. “Unconventional Medicine in the United States: Prevalence, Costs, and Patterns of Use.” New England Journal of Medicine 328, no. 4: 246–252. 10.1056/NEJM199301283280406. 8418405

[sjop70104-bib-0024] Ekor, M. 2014. “The Growing Use of Herbal Medicines: Issues Relating to Adverse Reactions and Challenges in Monitoring Safety.” Frontiers in Pharmacology 4: 177. 10.3389/fphar.2013.00177. PMC3887317 24454289

[sjop70104-bib-0025] Ernst, E. 2022. Alternative Medicine: A Critical Assessment of 202 Modalities. 2nd ed. Springer.

[sjop70104-bib-0100] Fischer, R. , and D. Boer . 2015. “Motivational Basis of Personality Traits: A Meta‐Analysis of Value‐Personality Correlations.” Journal of Personality 83, no. 5: 491–510. 10.1111/jopy.12125. 25142346

[sjop70104-bib-0026] Fjær, E. L. , E. R. Landet , C. L. McNamara , and T. A. Eikemo . 2020. “The Use of Complementary and Alternative Medicine (CAM) in Europe.” BMC Complementary Medicine and Therapies 20, no. 1: 1–9. 10.1186/s12906-020-02903-w. PMC7137515 32252735

[sjop70104-bib-0027] Frass, M. , R. P. Strassl , H. Friehs , M. Müllner , M. Kundi , and A. D. Kaye . 2012. “Use and Acceptance of Complementary and Alternative Medicine Among the General Population and Medical Personnel: A Systematic Review.” Ochsner Journal 12: 45–56. PMC3307506 22438782

[sjop70104-bib-0028] Furnham, A. , and J. Lovett . 2001. “Predicting the Use of Complementary Medicine: A Test of the Theories of Reasoned Action and Planned Behavior.” Journal of Applied Social Psychology 31, no. 12: 2588–2620. 10.1111/j.1559-1816.2001.tb00192.x.

[sjop70104-bib-0029] Galbraith, N. , T. Moss , V. Galbraith , and S. Purewal . 2018. “A Systematic Review of the Traits and Cognitions Associated With Use of and Belief in Complementary and Alternative Medicine (CAM).” Psychology, Health & Medicine 23, no. 7: 854–869. 10.1080/13548506.2018.1442010. 29468890

[sjop70104-bib-0030] Gale, N. 2014. “The Sociology of Traditional, Complementary and Alternative Medicine.” Sociology Compass 8, no. 6: 805–822. 10.1111/soc4.12182. PMC4146620 25177359

[sjop70104-bib-0031] Gordon, J. S. 1990. “Holistic Medicine and Mental Health Practice: Toward a New Synthesis.” American Journal of Orthopsychiatry 60, no. 3: 357–371. 10.1037/h0079185. 2200272

[sjop70104-bib-0032] Graham, K. D. , A. Steel , and J. Wardle . 2021. “The Intersection Between Models of Health and How Healing Transpires: A Metaethnographic Synthesis of Complementary Medicine Practitioners' Perceptions.” Journal of Alternative and Complementary Medicine 27, no. 7: 538–549. 10.1089/acm.2020.0521. 33877868

[sjop70104-bib-0033] Graham, K. D. , A. Steel , and J. Wardle . 2023. “The Converging Paradigms of Holism and Complexity: An Exploration of Naturopathic Clinical Case Management Using Complexity Science Principles.” Journal of Evaluation in Clinical Practice 29, no. 4: 662–681. 10.1111/jep.13721. 35703447

[sjop70104-bib-0034] Greene, J. A. , and J. Loscalzo . 2017. “Putting the Patient Back Together — Social Medicine, Network Medicine, and the Limits of Reductionism.” New England Journal of Medicine 377, no. 25: 2493–2499. 10.1056/NEJMms1706744. 29262277

[sjop70104-bib-0035] Grzywacz, J. G. , R. Neiberg , S. A. Quandt , W. Lang , R. A. Bell , and T. A. Arcury . 2012. “Measuring Differential Beliefs in Complementary Therapy Research: An Exploration of the Complementary and Alternative Medicine Beliefs Inventory (CAMBI).” Complementary Therapies in Medicine 20, no. 1–2: 54–60. 10.1016/j.ctim.2011.09.009. PMC3273715 22305249

[sjop70104-bib-0036] Guillaud, A. , N. Darbois , B. Allenet , and N. Pinsault . 2019. “Predictive Factors of Complementary and Alternative Medicine Use in the General Population in Europe: A Systematic Review.” Complementary Therapies in Medicine 42: 347–354. 10.1016/j.ctim.2018.12.014. 30670265

[sjop70104-bib-0037] Hansson, S. O. 2022. “Holism and pseudoholism.” In From Electrons to Elephants and Elections. Exploring the Role of Content and Context, edited by S. Wuppuluri and I. Stewart , 215–228. Springer. 10.1007/978-3-030-92192-7_13.

[sjop70104-bib-0038] Ho, E. Y. , C. L. Bylund , E. Wollney , E. B. Peterson , H. N. Wong , and C. J. Koenig . 2021. “A Systematic Review of Communication About Complementary and Integrative Health (CIH) in Global Biomedical Settings.” Patient Education and Counseling 104, no. 12: 2900–2911. 10.1016/j.pec.2021.05.015. 34030929

[sjop70104-bib-0039] Hoenders, R. , R. Ghelman , C. Portella , et al. 2024. “A Review of the WHO Strategy on Traditional, Complementary, and Integrative Medicine From the Perspective of Academic Consortia for Integrative Medicine and Health.” Frontiers in Medicine 11: 1395698. 10.3389/fmed.2024.1395698. PMC11201178 38933107

[sjop70104-bib-0040] Hughner, R. S. , and S. S. Kleine . 2004. “Views of Health in the Lay Sector: A Compilation and Review of How Individuals Think About Health.” Health: An Interdisciplinary Journal for the Social Study of Health, Illness and Medicine 8, no. 4: 395–422. 10.1177/1363459304045696. 15358896

[sjop70104-bib-0041] Hunter, J. , M. Leach , D. Chang , M. S. Lee , Y. Fei , and J. Liu . 2026. “Navigating Uncertainty in Evidence‐Informed Decision‐Making for Traditional, Complementary and Integrative Medicine: An Updated Critical Interpretive Review.” Integrative Medicine Research 15, no. 1: 101231. 10.1016/j.imr.2025.101231. PMC12974181 41816266

[sjop70104-bib-0042] Hyland, M. E. , G. T. Lewith , and C. Westoby . 2003. “Developing a Measure of Attitudes: The Holistic Complementary and Alternative Medicine Questionnaire.” Complementary Therapies in Medicine 11, no. 1: 33–38. 10.1016/S0965-2299(02)00113-9. 12667973

[sjop70104-bib-0043] Jensen, H. A. R. , and O. Ekholm . 2021. “Therapy‐Dependent Inconsistencies in Self‐Reported Use of Complementary and Alternative Medicine in the General Population: Findings From a Longitudinal Study.” BMJ Open 11, no. 11: e051647. 10.1136/bmjopen-2021-051647. PMC8628339 34836900

[sjop70104-bib-0044] Jütte, R. 2024. “Involving Voters and Consumers in Decision‐Making About the Health Care System–the Swiss Case: A Review.” Complementary Medicine Research 31, no. 1: 78–83. 10.1159/000534268. 37748443

[sjop70104-bib-0045] Karimi‐Dehkordi, M. , J. Spiers , and A. M. Clark . 2019. “An Evolutionary Concept Analysis of “Patients' Values”.” Nursing Outlook 67, no. 5: 523–539. 10.1016/j.outlook.2019.03.005. 31072646

[sjop70104-bib-0046] Kelner, M. , and B. Wellman . 1997. “Health Care and Consumer Choice: Medical and Alternative Therapies.” Social Science & Medicine 45, no. 2: 203–212. 10.1016/S0277-9536(96)00334-6. 9225408

[sjop70104-bib-0047] Kemppainen, L. M. , and T. T. Kemppainen . 2025. “Changes in Complementary and Alternative Medicine (CAM) Use From 2014 to 2023: Findings From a Cross‐National Population‐Based Survey in Europe.” Journal of Public Health. 10.1007/s10389-025-02494-1.

[sjop70104-bib-0048] Kristoffersen, A. E. , S. A. Quandt , and T. Stub . 2021. “Use of Complementary and Alternative Medicine in Norway: A Cross‐Sectional Survey With a Modified Norwegian Version of the International Questionnaire to Measure Use of Complementary and Alternative Medicine (I‐CAM‐QN).” BMC Complementary Medicine and Therapies 21, no. 1: 1–12. 10.1186/s12906-021-03258-6. PMC7962303 33726724

[sjop70104-bib-0049] Lechner, C. M. , C. Beierlein , E. Davidov , and S. H. Schwartz . 2024. “Measuring the Four Higher‐Order Values in Schwartz's Theory: Validation of a 17‐Item Inventory.” Journal of Personality Assessment 106, no. 5: 651–664. 10.1080/00223891.2024.2311193. 38408279

[sjop70104-bib-0050] Lee, E. L. , N. Richards , J. Harrison , and J. Barnes . 2022. “Prevalence of Use of Traditional, Complementary and Alternative Medicine by the General Population: A Systematic Review of National Studies Published From 2010 to 2019.” Drug Safety 45, no. 7: 713–735. 10.1007/s40264-022-01189-w. PMC9296440 35788539

[sjop70104-bib-0051] Lie, D. , and J. Boker . 2004. “Development and Validation of the CAM Health Belief Questionnaire (CHBQ) and CAM Use and Attitudes Amongst Medical Students.” BMC Medical Education 4: 1–9. 10.1186/1472-6920-4-2. PMC373452 14718061

[sjop70104-bib-0052] Lindeman, M. , and M. Verkasalo . 2005. “Measuring Values With the Short Schwartz's Value Survey.” Journal of Personality Assessment 85, no. 2: 170–178. 10.1207/s15327752jpa8502_09. 16171417

[sjop70104-bib-0053] Maghrabi, W. H. , H. Badr , A. Alkhyat , J. M. Schlaeger , and F. Cynthia . 2025. “Perceived Usefulness Drives the Use of Traditional, Complementary, and Integrative Medicine: A Systematic Umbrella Review.” Journal of Integrative and Complementary Medicine 31, no. 9: 768–777. 10.1089/jicm.2024.0841. 40372927

[sjop70104-bib-0054] Mazzocchi, F. 2012. “Complexity and the Reductionism‐Holism Debate in Systems Biology.” Wiley Interdisciplinary Reviews: Systems Biology and Medicine 4, no. 5: 413–427. 10.1002/wsbm.1181. 22761024

[sjop70104-bib-0055] McSwan, J. , J. Gudin , X. J. Song , et al. 2021. “Self‐Healing: A Concept for Musculoskeletal Body Pain Management–Scientific Evidence and Mode of Action.” Journal of Pain Research 14: 2943–2958. 10.2147/jpr.s321037. PMC8464648 34584448

[sjop70104-bib-0056] Messerli‐Rohrbach, V. 2000. “Personal Values and Medical Preferences: Postmaterialism, Spirituality, and the Use of Complementary Medicine.” Complementary Medicine Research 7, no. 4: 183–189. 10.1159/000021342. 11025393

[sjop70104-bib-0057] Ministry of Social Affairs and Health . 2009. Vaihtoehtohoitojen sääntelyn tarve: Vaihtoehtohoitoja koskevan lainsäädännön tarpeita selvittäneen työryhmän raportti. [Need for regulation of complementary and alternative therapies. Report of the Working Group to Examine the Need for Legislation on Complementary and Alternative Therapies]. Vol. 17. Reports of the Ministry of Social Affairs and Health. https://urn.fi/URN:ISBN:978‐952‐00‐2810‐7.

[sjop70104-bib-0058] Mora, D. C. , A. E. Kristoffersen , G. Overvåg , et al. 2022. “Safety of Complementary and Alternative Medicine (CAM) Treatment Among Children and Young Adults Who Suffer From Adverse Effects of Conventional Cancer Treatment: A Systematic Review.” Integrative Cancer Therapies 21: 15347354221105563. 10.1177/15347354221105563. PMC9218455 35726681

[sjop70104-bib-0059] National Center for Complementary and Integrative Health (NCCIH . 2021. Complementary, Alternative, or Integrative Health: What's in a Name? https://www.nccih.nih.gov/health/complementary‐alternative‐or‐integrative‐health‐whats‐in‐a‐name.

[sjop70104-bib-0060] Nazirova, Z. , and S. Borbala . 2024. “Values, Attitudes and the Behaviour Paradigm: A Systematic Literature Review.” Journal of Human Values 30, no. 2: 214–239. 10.1177/09716858241236902.

[sjop70104-bib-0061] Ng, J. Y. , T. Dhawan , E. Dogadova , et al. 2022. “Operational Definition of Complementary, Alternative, and Integrative Medicine Derived From a Systematic Search.” BMC Complementary Medicine and Therapies 22, no. 1: 104. 10.1186/s12906-022-03556-7. PMC9006507 35413882

[sjop70104-bib-0062] Ng, J. Y. , T. Dhawan , R. G. Fajardo , et al. 2023. “The Brief History of Complementary, Alternative, and Integrative Medicine Terminology and the Development and Creation of an Operational Definition.” Integrative Medicine Research 12, no. 4: 100978. 10.1016/j.imr.2023.100978. PMC10623279 37927333

[sjop70104-bib-0063] Ning, A. M. 2012. “How ‘Alternative’ Is CAM? Rethinking Conventional Dichotomies Between Biomedicine and Complementary/Alternative Medicine.” Health 17, no. 2: 135–158. 10.1177/1363459312447252. 22694916

[sjop70104-bib-0064] Oh, B. , P. Butow , B. Mullan , et al. 2010. “The Use and Perceived Benefits Resulting From the Use of Complementary and Alternative Medicine by Cancer Patients in Australia.” Asia‐Pacific Journal of Clinical Oncology 6, no. 4: 342–349. 10.1111/j.1743-7563.2010.01329.x. 21114784

[sjop70104-bib-0065] Park, C. L. , Z. E. Magin , and K. E. Gnall . 2026. “Individuals' Top Values and Health Behavior Engagement: Results of a National US Survey.” Psychology & Health 41, no. 3: 368–384. 10.1080/08870446.2024.2417945. PMC12018590 39445683

[sjop70104-bib-0066] Pincus, J. D. 2024. “Values as Motives: Implications for Theory, Methods, and Practice.” Integrative Psychological and Behavioral Science 58, no. 4: 1704–1750. 10.1007/s12124-024-09817-z. 38321258

[sjop70104-bib-0067] Politis, V. 2004. Routledge Philosophy Guidebook to Aristotle and the Metaphysics. Routledge. 10.4324/9780203647424.

[sjop70104-bib-0068] Posadzki, P. , L. K. Watson , and E. Ernst . 2013. “Adverse Effects of Herbal Medicines: An Overview of Systematic Reviews.” Clinical Medicine 13, no. 1: 7–12. 10.7861/clinmedicine.13-1-7. PMC5873713 23472485

[sjop70104-bib-0069] Publications of the Finnish Government . 2023. A strong and committed Finland. Programme of Prime Minister Petteri Orpo's Government 20 June 2023. Publications of the Finnish Government 2023: 60. https://urn.fi/URN:ISBN:978‐952‐383‐818‐5.

[sjop70104-bib-0101] Pyykkönen, M. , P. Aarva , S. Ahola , M. Pasanen , and K. Helin . 2023. “Use of Complementary and Integrative Health in Finland: A Cross‐Sectional Survey.” BMC Complementary Medicine and Therapies 23, no. 1: 279. 10.1186/s12906-023-04088-4. PMC10401804 37542306

[sjop70104-bib-0099] Quandt, S. A. , M. J. Verhoef , T. A. Arcury , et al. 2009. “Development of an International Questionnaire to Measure Use of Complementary and Alternative Medicine (I‐CAM‐Q).” Journal of Alternative and Complementary Medicine 15, no. 4: 331–339. 10.1089/acm.2008.0521. PMC3189003 19388855

[sjop70104-bib-0070] Ribeaux, P. , and M. Spence . 2001. “CAM Evaluation: What Are the Research Questions?” Complementary Therapies in Medicine 9, no. 3: 188–193. 10.1054/ctim.2001.0468. 11926434

[sjop70104-bib-0071] Rocca, E. , and R. L. Anjum . 2020. “Complexity, Reductionism and the Biomedical Model.” In Rethinking Causality, Complexity and Evidence for the Unique Patient. A CauseHealth Resource for Healthcare Professionals and the Clinical Encounter, edited by R. L. Anjum , S. Copeland , and E. Rocca , 75–94. Springer International Publishing. 10.1007/978-3-030-41239-5.

[sjop70104-bib-0072] Saad, J. M. , and J. O. Prochaska . 2020. “A Philosophy of Health: Life as Reality, Health as a Universal Value.” Palgrave Communications 6, no. 1: 45–45. 10.1057/s41599-020-0420-9. PMC7097380 32226633

[sjop70104-bib-0073] Saba, L. , and S. Tagliagambe . 2023. “Quantitative Medicine: Tracing the Transition From Holistic to Reductionist Approaches. A New “Quantitative Holism” Is Possible?” Journal of Public Health Research 12, no. 2: 22799036231182271. 10.1177/22799036231182271. PMC10286173 37361238

[sjop70104-bib-0074] Sagiv, L. , and S. Roccas . 2021. “How Do Values Affect Behavior? Let Me Count the Ways.” Personality and Social Psychology Review 25, no. 4: 295–316. 10.1177/10888683211015975. 34047241

[sjop70104-bib-0075] Sagiv, L. , S. Roccas , J. Cieciuch , and S. H. Schwartz . 2017. “Personal Values in Human Life.” Nature Human Behaviour 1, no. 9: 630–639. 10.1038/s41562-017-0185-3. 31024134

[sjop70104-bib-0076] Sagiv, L. , and S. H. Schwartz . 2022. “Personal values across cultures.” Annual Review of Psychology 73, no. 1: 517–546. 10.1146/annurev-psych-020821-125100. 34665670

[sjop70104-bib-0077] Schwartz, S. H. 1992. “Universals in the Content and Structure of Values: Theory and Empirical Tests in 20 Countries.” In Advances in Experimental Social Psychology, edited by M. Zanna , vol. 25, 1–65. Academic Press.

[sjop70104-bib-0078] Schwartz, S. H. 1994. “Are There Universal Aspects in the Structure and Content of Human Values?” Journal of Social Issues 50, no. 4: 19–45. 10.1111/j.1540-4560.1994.tb01196.x.

[sjop70104-bib-0079] Schwartz, S. H. 2012. “An Overview of the Schwartz Theory of Basic Values.” Online Readings in Psychology and Culture 2, no. 1: 11. 10.9707/2307-0919.1116.

[sjop70104-bib-0080] Schwartz, S. H. 2017. “The Refined Theory of Basic Values.” In Values and Behavior: Taking a Cross‐Cultural Perspective, edited by S. Roccas and L. Sagiv , 51–72. Springer International. 10.1007/978-3-319-56352-7_3.

[sjop70104-bib-0081] Sharif, I. , T. Yarash , F. Masood , R. M. Clifford , W. Davis , and T. M. Davis . 2020. “Complementary and Alternative Medicine Beliefs in Type 2 Diabetes: The Fremantle Diabetes Study Phase II.” Diabetes Research and Clinical Practice 166: 108311. 10.1016/j.diabres.2020.108311. 32673699

[sjop70104-bib-0082] Shroff, F. M. 2011. “Conceptualizing Holism in International Interdisciplinary Critical Perspective: Toward a Framework for Understanding Holistic Health.” Social Theory & Health 9: 244–255. 10.1057/sth.2011.6.

[sjop70104-bib-0083] Siahpush, M. 1999. “Why Do People Favour Alternative Medicine?” Australian and New Zealand Journal of Public Health 23, no. 3: 266–271. 10.1111/j.1467-842X.1999.tb01254.x. 10388170

[sjop70104-bib-0084] Sirois, F. M. , A. Salamonsen , and A. E. Kristoffersen . 2016. “Reasons for Continuing Use of Complementary and Alternative Medicine (CAM) in Students: A Consumer Commitment Model.” BMC Complementary and Alternative Medicine 16: 75. 10.1186/s12906-016-1059-3. PMC4765033 26911133

[sjop70104-bib-0086] Stub, T. , S. A. Quandt , T. A. Arcury , et al. 2016. “Perception of Risk and Communication Among Conventional and Complementary Health Care Providers Involving Cancer Patients' Use of Complementary Therapies: A Literature Review.” BMC Complementary and Alternative Medicine 16, no. 1: 353. 10.1186/s12906-016-1326-3. PMC5016861 27609097

[sjop70104-bib-0085] Stub, T. , S. A. Quandt , T. A. Arcury , J. C. Sandberg , and A. E. Kristoffersen . 2018. “Attitudes and Knowledge About Direct and Indirect Risks Among Conventional and Complementary Health Care Providers in Cancer Care.” BMC Complementary and Alternative Medicine 18, no. 1: 44. 10.1186/s12906-018-2106-z. PMC5793440 29386005

[sjop70104-bib-0087] Tangkiatkumjai, M. , H. Boardman , and D. M. Walker . 2020. “Potential Factors That Influence Usage of Complementary and Alternative Medicine Worldwide: A Systematic Review.” BMC Complementary Medicine and Therapies 20, no. 1: 1–15. 10.1186/s12906-020-03157-2. PMC7686746 33228697

[sjop70104-bib-0088] Taskinen, M. 2018. Short Schwartz's Value Survey–Kyselyn Uudelleenvalidointi Ja Suomalaiset Normit [Revalidation and Finnish Norms of the Short Schwartz's Value Survey Questionnaire]. University of Helsinki.

[sjop70104-bib-0089] Thomas, H. , M. Best , and G. Mitchell . 2020. “Whole‐Person Care in General Practice: The Nature of Whole‐Person Care.” Australian Journal of General Practice 49, no. 1/2: 54–60. 10.31128/ajgp-05-19-49501. 32008275

[sjop70104-bib-0090] Thomas, H. , G. Mitchell , J. Rich , and M. Best . 2018. “Definition of Whole Person Care in General Practice in the English Language Literature: A Systematic Review.” BMJ Open 8, no. 12: e023758–e023758. 10.1136/bmjopen-2018-023758. PMC6303638 30552268

[sjop70104-bib-0091] Vogt, H. , B. Hofmann , and L. Getz . 2016. “The New Holism: P4 Systems Medicine and the Medicalization of Health and Life Itself.” Medicine, Health Care, and Philosophy 19, no. 2: 307–323. 10.1007/s11019-016-9683-8. PMC4880637 26821201

[sjop70104-bib-0092] Wieland, L. S. , E. Manheimer , and B. M. Berman . 2011. “Development and Classification of an Operational Definition of Complementary and Alternative Medicine for the Cochrane Collaboration.” Alternative Therapies in Health and Medicine 17, no. 2: 50–59. PMC3196853 21717826

[sjop70104-bib-0093] Wode, K. , R. Henriksson , L. Sharp , A. Stoltenberg , and J. Hök Nordberg . 2019. “Cancer Patients' Use of Complementary and Alternative Medicine in Sweden: A Cross‐Sectional Study.” BMC Complementary and Alternative Medicine 19: 62. 10.1186/s12906-019-2452-5. PMC6417272 30866916

[sjop70104-bib-0094] World Health Organization (WHO) . 2013. WHO Traditional Medicine Strategy 2014–2023. World Health Organization (WHO). https://www.who.int/publications/i/item/9789241506096.

[sjop70104-bib-0095] World Health Organization (WHO) . 2020. Basic Documents. 49th ed. World Health Organization. https://apps.who.int/gb/bd/pdf_files/BD_49th‐en.pdf.

[sjop70104-bib-0096] World Health Organization (WHO) . 2025. Global Traditional Medicine Strategy 2025–2034. World Health Organization. https://www.who.int/teams/who‐global‐traditional‐medicine‐centre/traditional‐medicine‐strategy‐2025‐2034. 10.2471/BLT.25.293414 PMC12578523 41180270

[sjop70104-bib-0097] Wuppuluri, S. , and I. Stewart , eds. 2020. From Electrons to Elephants and Elections. Exploring the Role of Content and Context. Springer. 10.1007/978-3-030-92192-7.

[sjop70104-bib-0098] Zollman, C. , and A. Vickers . 1999. “What Is Complementary Medicine?” BMJ 319, no. 7211: 693–696. 10.1136/bmj.319.7211.693. PMC1116545 10480829

